# The consequences of subtracting the mean pattern in fMRI multivariate correlation analyses

**DOI:** 10.3389/fnins.2013.00174

**Published:** 2013-09-30

**Authors:** Lúcia Garrido, Maryam Vaziri-Pashkam, Ken Nakayama, Jeremy Wilmer

**Affiliations:** ^1^Vision Sciences Laboratory, Department of Psychology, Harvard UniversityCambridge, MA, USA; ^2^Department of Psychology, Wellesley CollegeWellesley, MA, USA

**Keywords:** fMRI, multivariate analyses, correlation analyses, subtraction mean pattern, cocktail-blank normalization

Multivariate pattern analyses of fMRI responses have become widely used in cognitive neuroscience. A popular method introduced by Haxby et al. (2001) is to correlate the patterns of responses to each condition across separate fMRI runs. These correlation analyses are applied both to (1) determine whether it is possible to discriminate/classify patterns of responses to two or more conditions, and (2) examine the relationships between patterns of responses to two or more conditions.

Before computing correlations between conditions, many researchers subtract each voxel’s overall mean response to all conditions from its response to each condition^1^. This is typically done independently for separate fMRI runs or datasets, for example even and odd runs. We refer to this step as “subtracting the mean pattern,” but it has also been called “normalization,” “subtraction of cocktail mean pattern,” or “cocktail blank normalization” (MacEvoy and Epstein, 2009; Op de Beeck, 2010).

Here, we discuss the effects of subtracting the mean pattern separately for two datasets^2^ in correlation analyses. Like other transformations of data, subtracting the mean pattern changes the relationships between conditions and therefore has consequences for results and their interpretation. Similar issues to the ones we discuss here have been described for the use of global signal covariates in univariate fMRI analyses (e.g., Aguirre et al., 1998), and more specifically in resting state analyses (e.g., Murphy et al., 2009; Saad et al., 2012). In fMRI multivariate correlation analyses, however, these changes in results and interpretation have been largely overlooked.

## Effects of subtracting the mean pattern on correlation matrices

In multivariate correlation analyses, researchers typically estimate the response at each voxel to each condition, for example, by using the general linear model to estimate regression coefficients. The response patterns across voxels are then used to calculate the correlations among conditions in a certain region of interest. It is common to have some voxels that have high or low absolute responses across all or some conditions, which may even be caused by noise. This creates a “common activation pattern” that is shared by some or all conditions and that similarly influences the magnitude of correlations among conditions (Sayres and Grill-Spector, 2008; Diedrichsen et al., 2011). In the attempt to avoid having the common activation pattern drive the correlations among response patterns, researchers subtract the mean pattern to obtain responses that are specific to each condition (Sayres and Grill-Spector, 2008; Op de Beeck, 2010). We argue, however that the mean pattern across all conditions is a poor estimate of this common activation pattern. Far from capturing only the component shared by all conditions, it is influenced by the signal specific to each condition. The mean pattern, therefore, does not isolate the common activation pattern. Rather, subtracting the mean codes the response to each condition in relation to all other conditions, and therefore introduces new dependencies between conditions that should be considered when interpreting the results.

**Figure 1:**
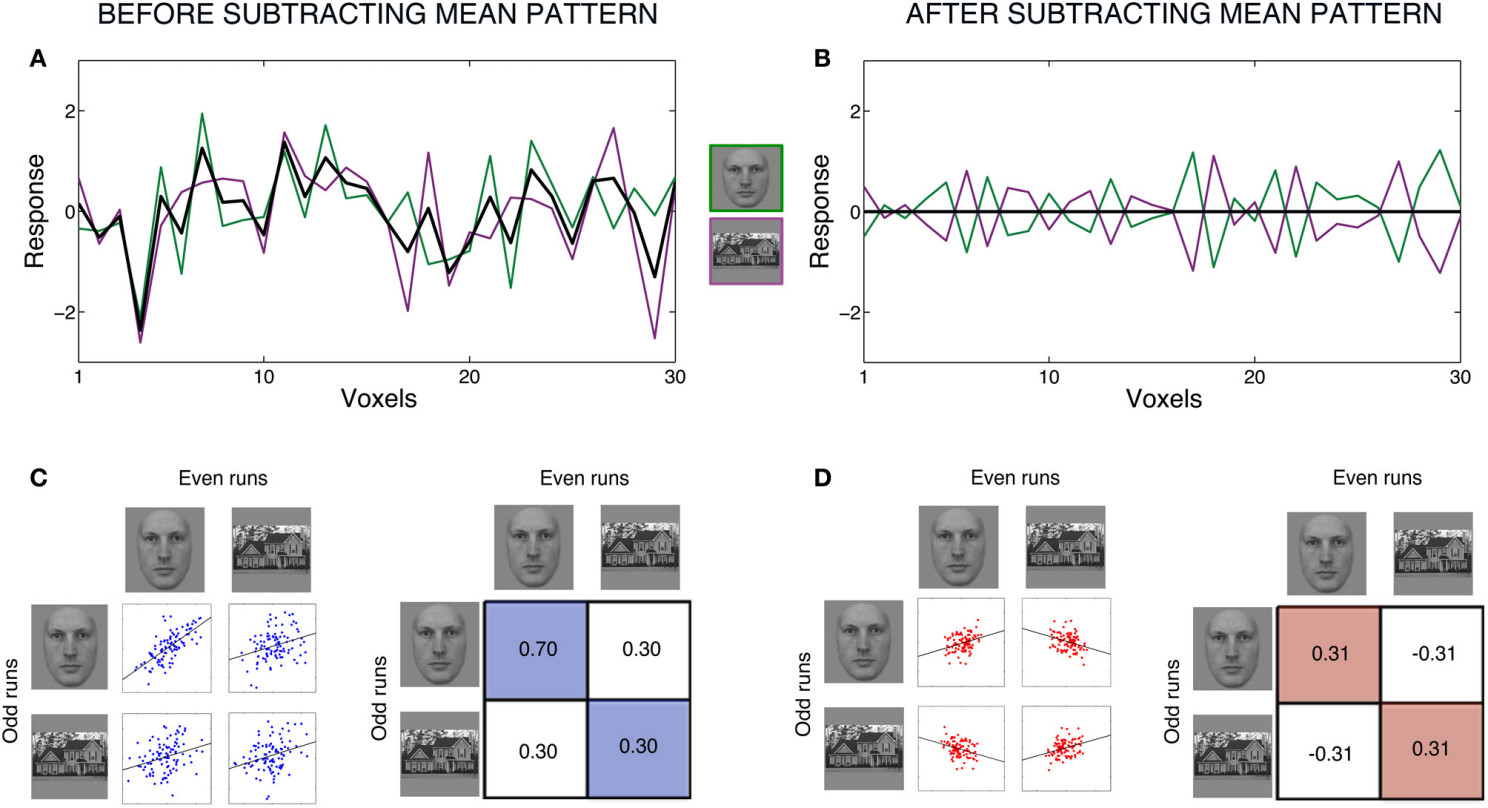
**Effects of subtracting the mean pattern for a case with two conditions**. Figure shows an example with simulated patterns of responses to two conditions, faces and houses. We simulated the responses for two datasets, which would correspond to average responses to each condition in even runs and average responses in odd runs. Responses were generated for 100 voxels. All correlations among conditions were set to be ~r = 0.3 to simulate the effects of a common activation pattern. In addition, the correlation for the condition faces between even and odd runs was set to be ~r = 0.7, to simulate moderately reliable response to this condition across datasets. We carried out many simulations but only retained the one in which the correlations were within 0.005 of the intended values. All data is for one single simulation (one “subject”). Left panels **(A,C)** show raw data (before subtracting the mean pattern) and right panels **(B,D)** show data after subtracting the mean pattern. Panels **(A,B)** show response patterns within half of the data (for example, even runs) for 30 voxels. Panel **(A)** shows the responses to each condition, with voxels on the x-axis and response magnitude on the y-axis. Faces are in green, houses are in purple, and the black line shows the mean of both conditions at each voxel. After subtracting the mean pattern from each condition, we obtain the patterns in Panel **(B)**. This plot illustrates the dependencies between conditions introduced by subtracting the mean pattern. The mean is now zero at each voxel, and therefore the response to faces at each voxel has the same magnitude but opposite sign as the response to houses. Furthermore, the two response patterns to faces and house after subtracting the mean pattern have the same variance and are perfectly anti-correlated. Panels **(C,D)** show scatter plots and correlation matrices among conditions *across* even and odd runs. Panel **(C)** show the original, simulated correlations between conditions. Panel **(D)** show what happens to correlations among conditions after subtracting the mean pattern, separately for even and odd runs. The correlation between faces in even runs and faces in odd runs decreases in magnitude. Moreover, the correlation between houses in even runs and houses in odd runs becomes positive. The correlations between faces and houses across runs become negative. These changes in correlations across datasets should be considered when interpreting the results and have consequences for further analyses using these correlations matrices.

To illustrate the consequences of subtracting the mean pattern, we simulated one example with patterns of responses to two conditions, faces and houses (Figure 1). We simulated the responses for two datasets, which would correspond to average responses to each condition in even runs and average responses in odd runs. The response patterns to faces were generated to be highly correlated across even and odd runs (*r* = 0.7). All other correlations were set to be approximately *r* = 0.3, to simulate the effect of a common activation pattern.

Figure 1A shows the raw data for each condition of one dataset, for example even runs, with voxels on the x-axis and response magnitude on the y-axis. We next explain all computations for one dataset, and they would be equally applied to the other dataset. Subtracting the mean pattern in Figure 1A corresponds to subtracting each voxel’s mean response across the two conditions (black line) from its response to each individual condition. After subtracting the mean pattern from each condition, we obtain the patterns in Figure 1B. The mean is now zero at each voxel. Note that, at each voxel, the response to each condition is now dependent on the other condition: a voxel’s response to one condition always has exactly the same magnitude but opposite sign as its response to the other condition. One clear way to see the creation of these dependencies is by looking at the correlations between patterns. In Figure 1A, the patterns of the two conditions had a correlation of *r* = 0.3, but in Figure 1B, the two conditions are perfectly anti-correlated, which will always be the case for two conditions. Furthermore, the response patterns to both faces and houses changed to become identical in variance.

This can be generalized to cases with any number of conditions. The response patterns across conditions for even runs can be represented in a *n*-by-*m* matrix **A**_even_, in which *n* is the number of conditions and *m* is the number of voxels. An element **A**_even_(*i,j*) corresponds to the response of condition *i* in voxel *j*. Therefore, each row of **A**_even_ contains the response pattern to a condition *i*. Subtracting the mean pattern corresponds to linearly combining the rows^3^ of **A**_even_ using matrix **G**:

(1) $$G=I_{n}-\frac{1}{n}$$

**G** is a *n*–by–*n* matrix and **I**_*n*_ is the *n*–by–*n* identity matrix. The final, transformed, data matrix is given by Equation 2:

(2) $$Y_{\text{even}}=GA_{\text{even}}$$

**Y**_even_ is the resulting data matrix for even runs and it has the same dimensions as **A**_even_.

Equation 2 shows that the response to each condition after subtracting the mean pattern is given by a linear combination of the responses to all the conditions. When we look at the variance of each condition after subtracting the mean pattern, we need to consider these same linear combinations—the variance of each condition is now distributed over all other conditions. These changes in response patterns of each condition within each dataset modify, in turn, the relationships between conditions *across* runs or datasets. These relationships correspond to the covariance or correlations between the rows of **Y**_even_ and the rows of **Y**_odd_ (**Y**_odd_ corresponds to the mean-pattern-subtracted matrix for odd runs and it is similarly derived using Equation 2).

The changes in correlations *across* datasets can be clearly seen in Figures 1C,D. Subtracting the mean pattern dramatically changes the absolute and relative values of the correlations among conditions. Whereas before subtracting the mean pattern (1C), the top left correlation (faces with faces) was the largest and all other correlations were equal, after subtracting the mean pattern (1D), the top left (faces with faces) and bottom right (houses with houses) correlations have converged to the same positive value, and the top right (faces with houses) and bottom left (houses with faces) correlations have decreased to the same negative value.

Critically, the correlation values have *different* interpretations before *vs.* after subtracting the mean pattern. The correlations in Figure 1C can be interpreted as the similarity or consistency of the response patterns to the two conditions across runs. More specifically, the correlation magnitudes before subtracting the mean pattern are influenced by signal shared between two conditions and the common activation pattern shared by all conditions (plus error). A higher correlation between the two patterns of responses to faces than between faces and houses indicates that the response patterns to faces share some specific signal that is not shared between faces and houses. This interpretation of correlation values is, however, no longer possible with the correlations in Figure 1D. A correlation between two conditions after subtracting the mean pattern is the correlation between two patterns *relative* to their respective means in the two separate datasets.

## Consequences for analyses that are based on correlation matrices

Correlation matrices are further used for other analyses, such as discrimination/classification analyses and Representational Similarity Analysis (RSA—Kriegeskorte et al., 2008a, b). Subtracting the mean pattern has consequences for the interpretation of these analyses.

In case of classification analyses, researchers compare the within-condition correlations with the between-condition correlations across even and odd runs to examine whether it is possible to discriminate the response patterns to two or more conditions. In Figure 1, if the within-condition correlations for faces and houses are higher than the between-condition correlations, we can conclude that we can discriminate between the response patterns to faces and houses. In this example, this is true for both the data before and after subtracting the mean pattern. In fact, as long as results are interpreted solely in terms of discrimination between conditions, subtracting the mean pattern does not affect these conclusions. In some cases, however, researchers have further interpreted their results as indicating that there is reliable signal or signal specific for a single condition in a region of interest (e.g., Haxby et al., 2001; Golarai et al., 2010; Weiner and Grill-Spector, 2010). This interpretation is no longer valid after subtracting the mean pattern. For example, in Figure 1D, the positive correlation between patterns for houses across even and odd runs cannot be interpreted as reliability across datasets. This should be taken into account when interpreting results after subtracting the mean pattern.

In case of RSA, researchers use correlations between conditions to examine how similar the representations of different conditions are in a certain region of interest, and thus characterize the information that is being represented. In certain cases, subtracting the mean pattern can change the relative magnitudes of correlation between conditions, which will result in changes in the rank-order of correlations of pairs of conditions, and have consequences for RSA results. This is more likely to happen if one or more conditions have large variances compared to other conditions, or if one or more pair of conditions have large covariance compared with the covariance of other pairs of conditions. In all cases, subtracting the mean pattern substantially obscures interpretation and understanding of these analyses. Conversely, correlation values before subtracting the mean pattern straightforwardly indicate which conditions share more signal than others.

Finally, some researchers have interpreted negative correlations as opposing patterns of activity for the conditions (Hanson et al., 2004; Weiner and Grill-Spector, 2010). Negative correlations, however, happen just because of the transformations that occur with subtracting the mean pattern, given that all correlations average to approximately zero (Figure 1D). Therefore, anti-correlations are not neurally meaningful (see Murphy et al., 2009).

## Conclusions

We suggest that a suitable approach to the various problems created by subtracting the mean pattern is to skip this step, and work instead with the original data; this approach enables most common analyses. There might be cases, nevertheless, in which it is important to accurately estimate and remove the influence of a common activation pattern. Examples of these cases are when the covariance between all conditions is extremely high, or if researchers want to compare correlation magnitudes across regions^4^. Recently, Diedrichsen et al. (2011) proposed a novel method to estimate the true correlations between response patterns using a pattern-component model, and this method might be particularly useful for these cases.

To conclude, subtracting the mean pattern changes the relationships between conditions. Here, we described how this step, applied to two separate datasets, changes the variance of each condition, and the relative correlations between conditions *across* datasets. Consequently, subtracting the mean pattern changes the correlation matrices that are the starting point for multivariate correlation analyses. Critically, we showed that subtracting the mean pattern *always* constrains interpretations of those correlations and that, after subtracting the mean pattern, correlations should not be interpreted as similarity, consistency, reproducibility, or reliability of pairs of response patterns across runs or datasets. We think that comprehending these changes can lead to a broader understanding of the consequences of subtracting the mean pattern in correlation analyses.

## Footnotes

^1^Examples of studies that subtracted the mean pattern are: Pietrini et al. (2004); Williams et al. (2007), (2008); Sayres and Grill-Spector (2008); Howard et al. (2009); Chan et al. (2010); Golarai et al. (2010); Kravitz et al. (2010), (2011); Op de Beeck (2010); Op de Beeck et al. (2010); Weiner and Grill-Spector (2010); Weiner et al. (2010); Golomb and Kanwisher (2012). In some of these studies, the authors also divided the subtracted mean pattern by the variance of responses. This additional step does not affect the issues discussed in this manuscript. The papers by Chan et al. (2010) and Kravitz et al. (2010) did not explicitly mention subtracting the mean pattern, yet the authors confirmed that the published figures and results were based on data for which the mean pattern had been subtracted (pers. commun.).

^2^Here, we use the term “dataset” to refer to half or part of the data to which subtraction of mean pattern is applied. For example, data from even and odd runs form two datasets. Data from different runs, sessions or experiments is also considered to be separate datasets. The correlation analyses described here are computed across datasets.

^3^We note that subtracting the mean pattern results in having the mean of each *column* of **A**_even_ equal to zero. Conversely, by using Pearson correlation, we are also automatically mean centering the *rows* of **A**_even_.

^4^Note that this is not possible with raw data, given that different levels of common activation pattern across regions influences those correlations, nor it is possible after subtracting the mean pattern.
